# The effect of sodium and magnesium ions on the properties of calcium–phosphate biomaterials

**DOI:** 10.1007/s40204-019-0117-6

**Published:** 2019-05-24

**Authors:** Ekaterina Lyutova, Lyudmila Borilo, Elena Izosimova

**Affiliations:** National Research Tomsk State University, Tomsk, Russia

**Keywords:** Calcium–phosphate materials, Biomaterial, Sol–gel synthesis

## Abstract

A calcium–phosphate system was obtained by sol–gel method from 0.4 M solutions based on ethyl alcohol, tetraethoxysilane, phosphoric acid, calcium nitrate, and magnesium nitrate, sodium chloride. Compositions with different contents of CaO, Na_2_O, and MgO were prepared. After maturation of the solutions, heat treatments were applied at 60 °C for 30 min; and followed by 600 °C and 800 °C for 1 h. Solution with 20 wt% MgO was found suitable for film production. The physicochemical processes of the formation of materials were studied, including the main stages: removal of physically bound and chemically bound water, combustion of alcohol and the products of thermo-oxidative destruction of ethoxy groups, and crystallization processes. The phase composition and structure of the films obtained were established at 600 °C and above when crystalline forms of SiO_2_, CaSiO_3_, Ca_2_P_2_O_7_, and complex phosphates were fixed. In the system with the addition of magnesium ions, β-cristobalite SiO_2_ and stenfieldt Mg_3_Ca_3_(PO_4_)_4_ were detected; however, a crystalline sample could only be obtained at 800 °C. In the system with sodium ions, chemical compounds Ca_5_(PO_4_)_3_Cl, NaCl, and SiO_2_ were determined. A uniform film coating was formed on the surface of the substrate. The introduction of sodium oxide into the SiO_2_–P_2_O_5_–CaO system increased the bioactivity of the materials obtained.

## Introduction

The tasks of modern medicine and biotechnology include not only the creation of implants, the replacement of bone tissues and organs, but also the synthesis of biologically active materials that contribute to the most complete tissue repair and the maintenance of necessary body functions (Barinov 2010; Dorozhkin 2010; Evdokimov et al. 2014; Bagherpour et al. 2018; Komlev et al. 2012). The bone system of a living organism is formed and maintained by complex biochemical reactions (Barinov 2010; Dorozhkin 2010). The main elements of these reactions are Ca, P, O, H, Na, and Mg.

It is known that calcium is one of the important elements for a living organism: its cations control the transport of inorganic ions and organic substances through cell membranes in the exchange process associated with the entry and exit of reaction products from the cell (Vallet-Regi and Gonzalez-Calbet 2004; Dorozhkin 2016; Barinov and Komlev 2014a, b).

Around the world, calcium hydroxyphosphates are intensively studied and produced (Barinov and Komlev 2014a, b, 2016).

It is well known that when bone calcium hydroxyphosphate (hydroxyapatite) is introduced into the body as a result of slow resorption in the body and involvement in the metabolic process, osteogenesis improves, but calcium hydroxyphosphate does not possess transdermal ability (Narayanan et al. 2008).

In addition, the system containing SiO_2_–CaO–P_2_O_5_–Na_2_O has a higher biological activity compared to hydroxyapatite. Magnesium is another key element that increases bioactivity. Magnesium plays a significant role in stimulating osteoblastic cells and reduces bone resorption (Narayanan et al. 2008). The substitution of magnesium ion in the silicate skeleton affects the structure and properties of the bone. On the other hand, the magnesium ion proceeds as a silicate network modifier (Pet’kov et al. 2006; Popa et al. 2017).

There are numerous ways to obtain modified calcium–phosphates, the essence of which is to be precipitated from the salts, calcium oxide or hydroxide using orthophosphoric acid or one and two substituted phosphate salts, followed by hydrolysis in solution, under hydrothermal conditions or as a result of pyrolysis (Pet’kov et al. 2006; Popa et al. 2017; Kukueva et al. 2017).

However, materials based on hydroxyapatite are currently of limited use, due to its low solubility (and as a result, resorbability) in body fluids as well as its high fragility.

In recent years, calcium–phosphate coatings are widely used in medicine (Matsumoto et al. 2009; Komlev et al. 2012; Vallet-Regi and Gonzalez-Calbet 2004).

Various methods are used in the development and formation of biocoatings (Jmal and Bouaziz 2017; Vijayalakshmi and Rajeswari 2006; Chrysafi and Perraki 2007); the most promising is the sol–gel method of obtaining biomaterials, since it provides high purity of products, the ability to regulate the chemical composition and reduces energy costs. Using the sol–gel method allows you to influence the surface properties of materials, which is especially important for implants, since the interaction with the body occurs through the surface.

The most progressive attempt to solving the problems of regenerative medicine is to acquire composite calcium–phosphate coatings (Komlev et al. 2012).

It is proved that the presence of silicon in the volume of calcium–phosphate material and on its surface accelerates the fusion of the implant with the bone (osteointegration) (Popa et al. 2017; Kukueva et al. 2017; Moghanian et al. 2018; Vijayalakshmi and Rajeswari 2006; Chrysafi and Perraki 2007; Jmal and Bouaziz 2017).

Silicate groups in the calcium–phosphate system significantly increase the rate of osteogenesis in vivo after implantation. The process of bone tissue remodeling occurs about twice faster on silicon-containing hydroxyapatite (SiHA) than on hydroxyapatite in the absence of silicon (Petrovskaya et al. 2016; Bjornoy et al. 2016).

Partial replacement of phosphate groups by silicate reduces the size of crystallites and changes the structure of the grains of calcium–phosphate material, increases its dissolution rate in body fluids, thereby accelerating the remodeling process according to the mechanism of deposition from extracellular fluids (Borilo et al. 2016).

Thus, the purpose of this study was to establish the effect of the addition of Na^+^ and Mg^2+^ ions on the properties of thin-film materials obtained on the basis of the SiO_2_–P_2_O_5_–CaO system by sol–gel method.

## Experimental

### Materials and methods

#### Source reagents

Ethyl alcohol (96%, Russia), tetraethoxysilane (puriss. spec., Germany), orthophosphoric acid (puriss. spec., Himmed Russia.), calcium chloride (p.a., Himmed Russia), sodium chloride (p.a., Himmed Russia), magnesium nitrate (p.a., Himmed Russia).

#### Synthesis of materials

Particular attention should be paid to the choice of precursors in the preparation of materials using sol–gel technology (Petrovskaya et al. 2016). The required viscosity for obtaining materials is achieved by choosing the optimal ratio of components in the solution. It is known that coatings based on calcium–phosphate are used to replace damaged bone tissue and they are characterized by high biocompatibility and biological activity with a Ca/P ratio in the range of 1.67 (Vallet-Regi and Gonzalez-Calbet 2004).

An aggregated stable gel was prepared to obtain a biologically active thin-film material.

For the system SiO_2_–P_2_O_5_–CaO (composition 1), solutions were prepared by dissolving calcium chloride (34 wt%) in a solvent (ethyl alcohol), followed by the addition of tetraethoxysilane (52 wt%) and orthophosphoric acid (14 wt%). The solution system SiO_2_–P_2_O_5_–CaO–NaO (composition 2) was prepared the same way as composition 1, but with the addition of sodium chloride (5–20 wt%) due to the content of calcium oxide in the system, and the amount of tetraethoxysilane remained unchanged. The solution system SiO_2_–P_2_O_5_–CaO–MgO (composition 3) was prepared in the same way as composition 1, with the addition of magnesium nitrate (5–20 wt%) due to the calcium oxide content of the system, and the amount of tetraethoxysilane remained unchanged. The aging of the sol was carried out at room temperature for 3 days.

#### Characterization techniques

Film-forming solutions were kept in a thermostat at a temperature of 25 °C. Films were obtained on monocrystalline silicon substrates (model substrate) by centrifuging at a centrifugal speed of 3000 rpm, followed by heat treatments at 60 °C for 20 min and at 600 °C for 1 h. To study the film-forming ability of the solutions, their viscosity was measured using a glass viscometer (with a capillary diameter of 0.99 mm, at a temperature of 25 °C).

Infra-red spectra of powders and solutions were done using Fourier infra-red spectrometer Nicolet 6700 within frequency domain 400–4000 cm^−1^. Thermal analysis was performed simultaneously by a TGA/DSC/DTA analyzer STA 449C Jupiter at linear heating to 1000 °C (heating speed 10 °C/min) in airflow 100 mL/min.

Activation energy of each stage of thermal destruction of samples was defined with the help of approximation methods by thermographic and thermogravimetric measurements using the Horowitz–Metzger equation. X-ray phase analysis was performed using diffractometers Rigaku MiniFlex 600 and Shimadzu XRD-6000 (CuKα radiation in the range of 2θ 3–120 °C step size 2θ 0.01–0.02 °C and time per step 0.3–0.5 °C) using database PCPDFWIN and JSPDS, and program of full-profile phase analysis of Powder Cell 2.4 Structure (scanning electronic microscopy, SEM) and chemical composition (energy-dispersive X-ray spectroscopy, EDX) of samples were analyzed on a Hitachi TM-3000 scanning electronic microscope with accessory Quantax-70 for energy-dispersive microanalyses.

Evaluation of the bioactivity of the obtained materials was studied in vitro by keeping the samples in a cell-free simulation of simulated body fluid (SBF) for 14 days. Samples were placed in a model environment SBF at a constant temperature of 37 ± 0.5 °C and pH 7.4. The solution was changed every day for 14 days. The composition of the SBF solution is described in the literature (Borilo et al. 2016; Kokubo et al. 1990).

## Results and discussion

The stability of film-forming solutions (FFS) in time is important for the production of films. Viscosity was taken as a criterion for the film-forming ability of solutions.

It was established that for composition 1, the lifetime of the solutions lasted for 7 days. In the first 2 days, there was a sharp increase in viscosity to 3.8 mm^2^/s, and then, the viscosity changed slightly.

With the introduction of sodium ions into the system by more than 10 wt% solution, on the 2nd day, a gelation was observed, which became unsuitable for the production of films. When sodium ions at 10 wt% were added into the system, the solutions were considered suitable for producing films within 7 days. On the 1st day, a sharp increase in viscosity was not observed, though there was a slight increase by 2.3 mm^2^/s, but on the 7th day, viscosity reached a value of 6.1 mm^2^/s.

It was established that with the introduction of magnesium ions into the system with 5–20 wt% content on the 1st day for solutions, the viscosity displayed a value in the range from 1.72 to 1.82 mm^2^/s depending on the composition of the solution. On the 2nd day, a significant decrease in viscosity was observed in solutions with a magnesium ion content of 5–15 wt%, and on the 3rd day a fine white precipitate was deposited. The solution containing magnesium ions was stable for 13 days (Table 1).

**Table 1:** Viscosity values for time dependent film-forming solutions, mm^2^/s

Composition of FFS	Storage time of FFS (days)				
	0	2	5	7	13
System SiO_2_–P_2_O_5_–CaO (composition 1)	1.3	3.8	4.2	4.4	–
System SiO_2_–P_2_O_5_–CaO–NaO (composition 2)	2.0	2.3	3.5	6.1	–
System SiO_2_–P_2_O_5_–CaO–MgO (composition 3)	1.7	1.8	1.7	1.8	1.8

The obtained patterns of time-based viscosity change allowed us to conditionally distinguish three stages in the system of solution processes. At the first stage, hydrolysis and polycondensation processes occurred in the solution, which led to the formation of a molecular network, which entailed a sharp change in viscosity values. When sodium ions were added to the system, the viscosity on the 1st day changed slightly from 2 to 2.3 mm^2^/s, and with the introduction of magnesium ions, there was a slight increase to 1.8 mm^2^/s.

Then, the processes in the solutions were slowed down, the hydrolysis and polycondensation reactions continued, but proceeded at a low speed, due to spatial difficulties. After accumulation of tetra- and pentasiloxanes with –OH terminal groups in solution, the viscosity increased through the cyclization of siloxanes due to the mobility of the Si–O bond. The presence of H_3_PO_4_ content in the system accelerated the processes of hydrolysis and condensation by increasing the acidity of the medium. Ca^2+^, Mg^2+^, and Na^+^ cations seemed capable of forming poorly soluble compounds which contributed to the dehydration of the surface layer. However, the introduction of magnesium ions into the system slowed down the processes in solution. The introduction of electrolytes led to the acceleration of polycondensation, and also contributed to the reduction of aggregative stability of the colloidal system and accelerated the process of sol to gel transition. In this period, the solutions were no longer suitable for the production of films. It was established experimentally that when the viscosity of film-forming solutions was over 4.4 mm^2^/s, the films were uneven and peeled off from the substrate surface.

For compounds 1 and 2, the solutions seemed suitable for producing films within 7 days, the introduction of magnesium oxide into the system led to an increase in the time interval to 14 days, due to the slowing down of the crosslinking of three-dimensional structures (Jmal and Bouaziz 2017). In Table 2, the IR spectra of the solutions are presented.

**Table 2:** FTIR spectrometry data for solutions

Oбpaзeц			Vibrations (type)
Composition 1	Composition 2	Composition 3	
3326.7	3327.5	3322.9	Adsorbed water (valence vibrations OH)
2976.2; 2879.4	2976.5	2974.4; 2875.6	Valence vibrations of C–H
2928.9	2928.7	2927.8	Valence vibrations of –CH_2_–
1654.1	1649.2	1649.3	Deformation vibrations of water
1446.5; 1410.9 1383.9	–	1453.7; 1418.3 1373.5	Deformation vibrations of CH_2_, CH_3_
1319.6	1319.5	1328.8	Deformation oscillations –CH_2_–
1271.2	1271.8	1274.7	Deformation oscillations –OH in primary alcohols
1088.4; 1045.5	1088.4; 1042.6	1088.4; 1041.8	Valence vibrations P=O, –PO_4_^3−^ group
800.4	798.3	795.8	δ(Si–O–Si)
–	–	877.8	Stretching vibrations of Si–O–H, Mg–O–Mg
603.7	603.4	601.9	Deformation oscillations of Si–O–, P–O–P
428.8	429.6	436.1	[CaO_6_]
–	423,4	–	O–Na–O

The peaks in the range of 3322–3326 cm^−1^ corresponded to the vibrations of –OH-free groups. Within the range 2874–2927 cm^−1^, the valence vibrations of –CH_3_ and –CH_2_– were recorded. The valence vibrations of the P=O, –PO_4_^3–^ groups corresponded to 1041–1045, 1088 cm^−1^. In the range of 795–800 cm^−1^, the valence vibrations of Si–O–Si were fixed, and in the range of 877 cm^−1^, the valence vibrations of Si–O–H, though the Mg–O–Mg bonds were also fixed in the same range.

In the range of 428–436 cm^−1^, deformation Ca–O– vibrations were fixed; as a result, the ≡Si–O–PO_2_–O– chains were crosslinked with calcium cations in stabilizing the silicate gel and forming a three-dimensional structure. The deformation vibrations of the Si–O–, P–O–P oscillations correspond to vibrations in the range of 601–603 cm^−1^ (Moghanian et al. 2018).

According to the thermal analysis of the dried solutions and the X-ray phase analysis of the products of their thermal destruction, it was possible to establish the formation processes of oxide systems. The method of thermal analysis determined the optimal synthesis temperature, and X-ray phase analysis indicated the composition of substances formed during the thermolysis of dried film-forming solutions.

**Fig. 1:**
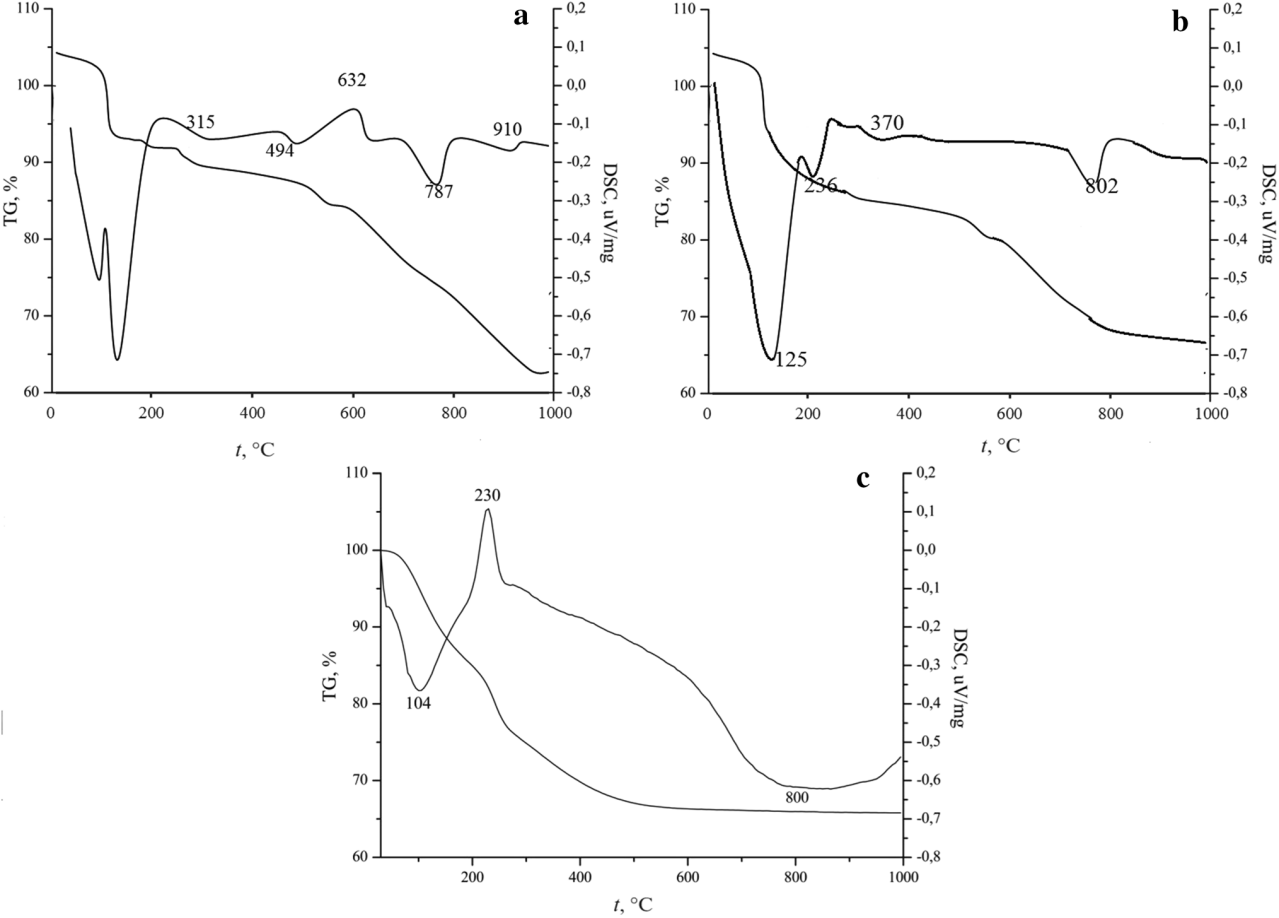
Thermal analysis results: **a** composition 1; **b** composition 2; **c** composition 3

Regardless of the composition of the system, the materials’ formation process took place in three stages. The data presented in Fig. 1 show that the thermal decomposition of the sample is accompanied by a sharp drop in the mass of the sample (15%) before 400 °C.

In this area, the physical and chemical bound water is removed, accompanied by three endothermic effects for the SiO_2_–P_2_O_5_–CaO system at temperatures of 102, 124, and 315 °C, one endothermic effect and an exothermic effect for the SiO_2_–P_2_O_5_–CaO–MgO system at temperatures of 104, 230 °C and for the SiO_2_–P_2_O_5_–CaO–NaO system at temperatures of 125 and 236 °C. The activation energy of each stage of material formation has been calculated by the Metzger–Horowitz equation (Petrovskaya et al. 2016; Petrović et al. 2001).

The value of the activation energy (*E*_a_) of the process occurring in the considered temperature range is 33–35 kJ/mol. Such a low value of *E*_a_ confirms the destruction of intermolecular bonds. Furthermore, in the temperature range of 400–600 °C, alcohol and thermo-oxidative decomposition products of ethoxy groups are burned and the sample mass changes slightly for the first and second compounds, and in the presence of magnesium in the system, the second stage is accompanied by a large drop in mass without temperature effects. In the last stage, at temperatures above 700 °C, crystallization and polymorphic transformations of oxides occur in the system. The activation energy of the process occurring in this temperature range is 120–160 kJ/mol, which is characteristic for the destruction of chemical bonds in the compounds.

**Fig. 2:**
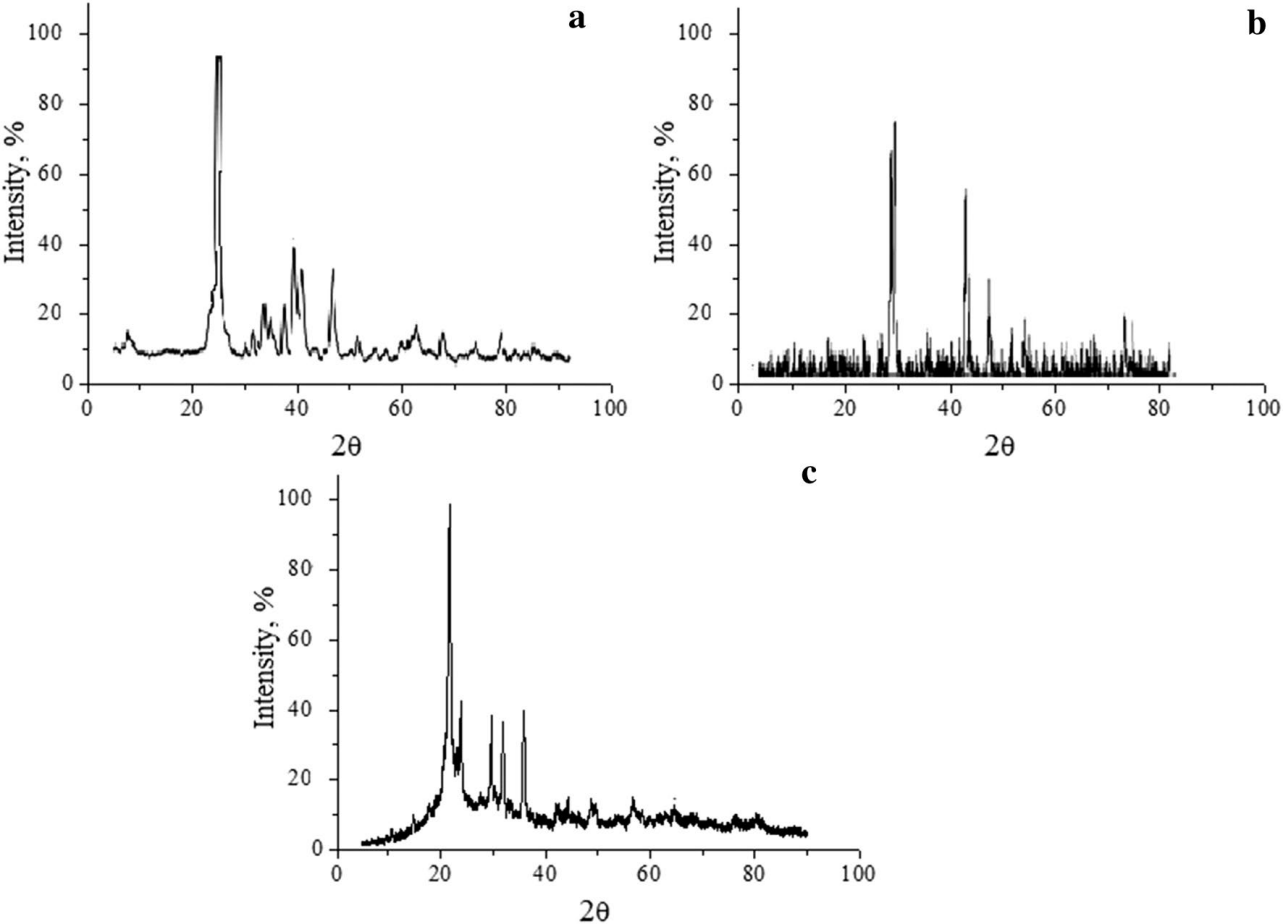
XRD patterns: **a** composition 1; **b** composition 2; **c** composition 3

The identification of the phases present in a polycrystalline sample was carried out using high-quality X-ray phase analysis (Fig. 2). For the SiO_2_–P_2_O_5_–CaO system, β–Ca_2_P_2_O_7_—β-calcium pyrophosphate, Ca_5_(PO_4_)_3_Cl—chloroapatite, α-Ca_2_SiO_4_—α-calcium orthosilicate, CaSiO_3_—wollastite, and SiO_2_—(quartz) crystalline are fixed at a temperature of 600 °C (Fig. 2a).

For the SiO_2_–P_2_O_5_–CaO–MgO system, the samples at 600 °C are amorphous. Therefore, it is necessary to increase the temperature treatment to 800 °C. β-Cristobalite SiO_2_ and Mg_3_Ca_3_(PO_4_)_4_ stenfieldt were found (Fig. 2c). For the system SiO_2_–P_2_O_5_–CaO–NaO at 600 °C, chemical compounds Ca_5_(PO_4_)_3_Cl, NaCl, and SiO_2_ are determined (Fig. 2b).

**Fig. 3:**
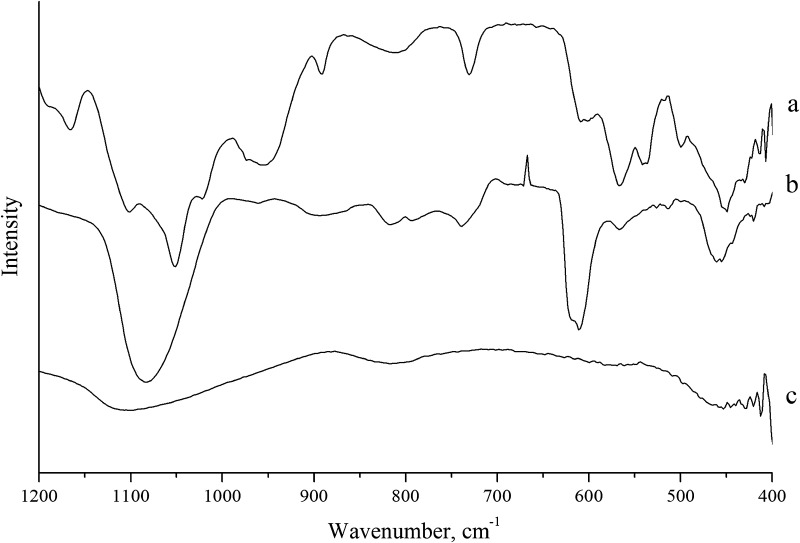
IR spectrometry results for materials annealed at 600 °C: a—composition 1; b—composition 2; c—composition 3

**Fig. 4:**
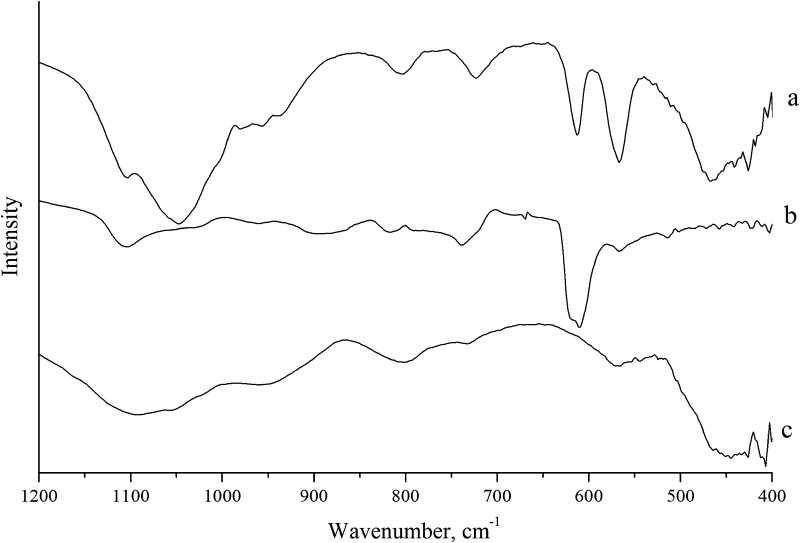
IR spectrometry results for materials annealed at 800 °C: a—composition 1; b—composition 2; c—composition 3

The results of IR spectroscopy of samples’ processing at different temperatures at 600 and 800 °C (Table 3, Figs. 3, 4) confirm the results of thermal analysis and X-ray diffraction analysis.

**Table 3:** IR spectroscopy results of films obtained from FFS at different annealing temperatures

Presence of bands in IR spectra, cm^−1^ (at various temperatures, °C)						Vibrations (type)
Composition 1		Composition 2		Composition 3		
600	800	600	800	600	800	
1040.1 929.4	1078.0 965.5	1083.0 960.8	1104.7 959.8	1084.7	1069.7 941.2	Valence vibrations P=O, –PO_4_^3−^ group
–	–	–	–	870.3	870.4	Mg–O–Mg
776.4 680.9	788.5 681.3	739.0	738.7 669.8	803.2	790.2	δ(Si–O–Si)
622.3 592.3	619.3 610.2	610.7	610.4	–	–	[SiO_4_]
584.1 547.7 501.8	566.6 516.2 502.0	566.8 513.9	566.9 514.5 501.7	560.1	555.4	Deformation oscillations of Si–O–
472.9 458.3	472.9 457.8	455.1	472.2 457.9	443.6	438.0	[CaO_6_]
–	–	420.3	423.4	–	–	O–Na–O

The presence of Si–O–Si valence vibrations and Si–O– deformation vibrations shows that these bonds are not destroyed at high temperatures (Kondratowicz 2007; Yashima et al. 2003; Zhang et al. 2013; Letaпef et al. 2014).

At temperatures of 600 (Fig. 3) and 800 °C (Fig. 4), the structure of the material is formed by silicon–oxygen and phosphorus–oxygen atomic groups, as evidenced by the presence in the IR spectrum of bands characteristic of valence vibrations P=O, –PO_4_^3–^, deformation Si–O vibrations, δ (Si–O–Si). The absorption bands in low-frequency region of 400–473 cm^−1^ are associated with vibrations of calcium with oxygen bonds in [CaO_6_]—octahedra and vibrations of the O–Na–O, Mg–O–Mg bond.

**Fig. 5:**
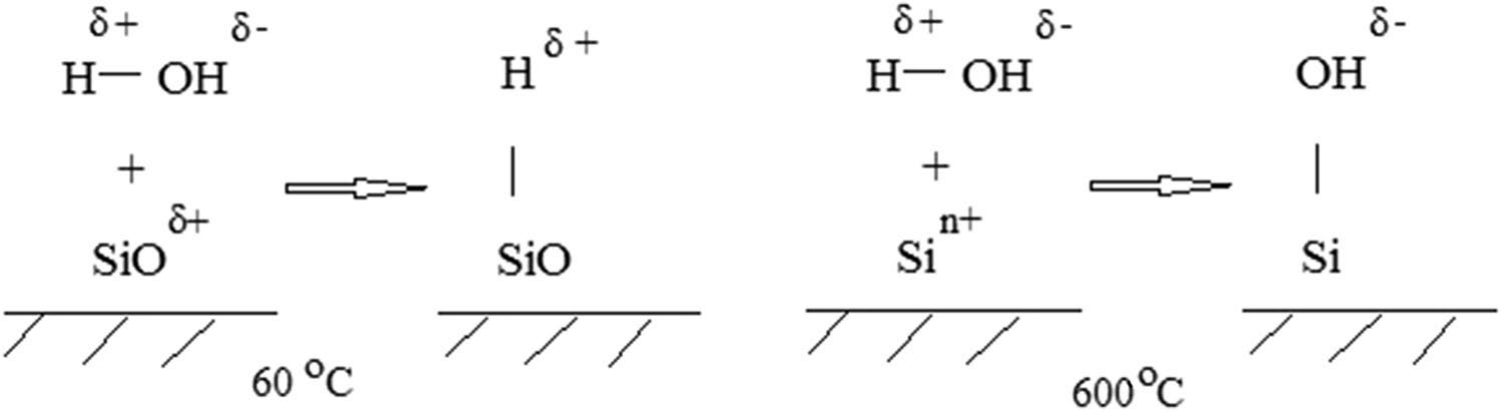
Mechanism of interaction of water molecules with different centers on the surface of material

Bioactive properties depend on the charge and porosity of the surface of materials. The studies in this work are focused on acid–base properties of an FFS surface dried at 60 °C and annealed at 600 °C. Regardless of the composition of the initial system, the Brønsted acid sites, characterized by pH 4, predominate on the surface of samples dried at 60 °C. The adsorption mechanism is shown in Fig. 5. For samples annealed at 600 °C, the pH value increases sharply to 10, which indicates that the fine sample is a Brønsted base; the adsorption mechanism is shown in Fig. 5. This surface charge affects the distribution of ions around it when immersed in SBF.

**Fig. 6:**
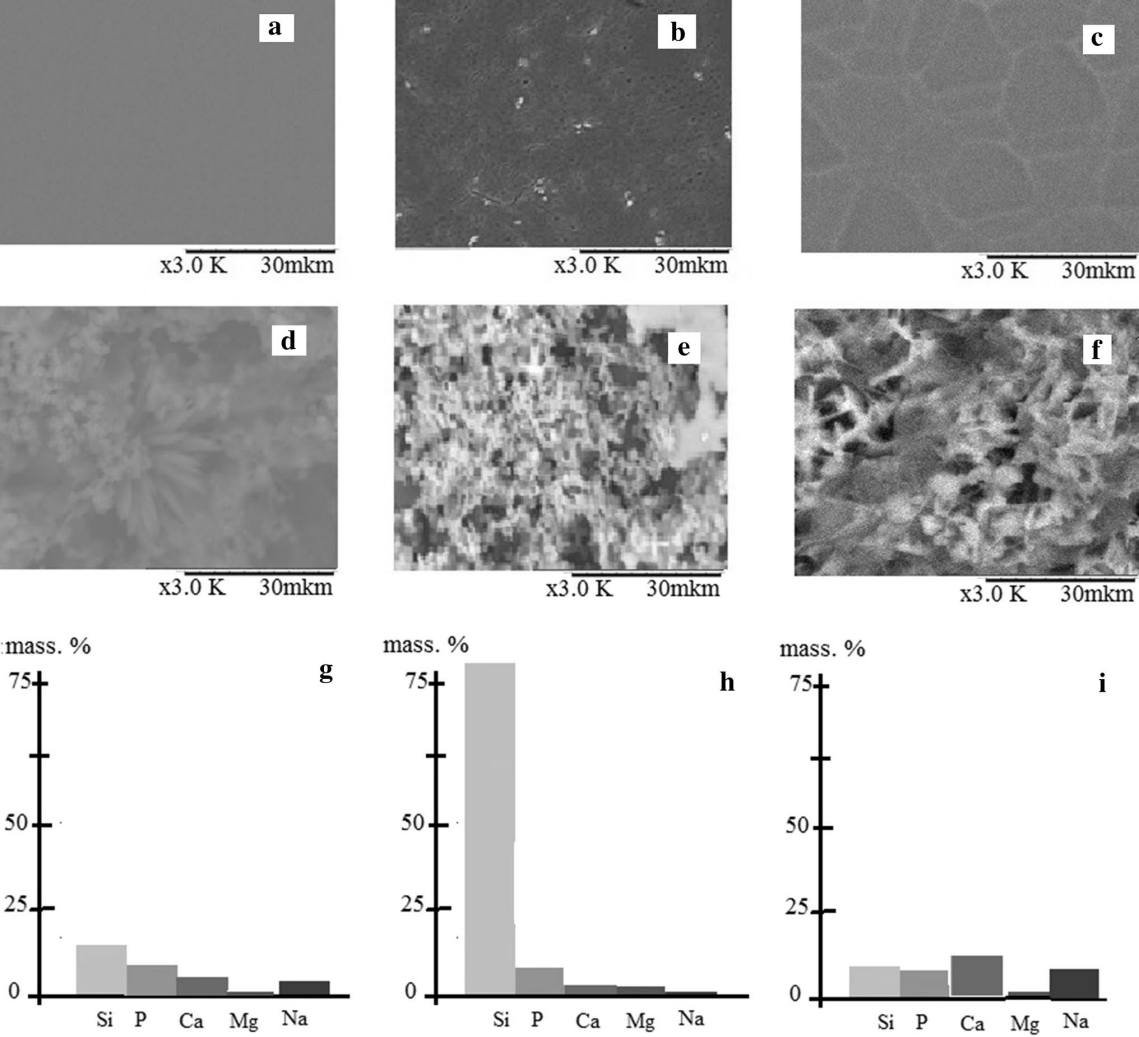
SEM images of films before (**a**–**c**) and after soaking in SBF (**d**–**f**), concentration ions is observed after immersion in SBF (**g**–**i**): **a**, **d**, **g** composition 1; **b**, **e**, **h** composition 2; **c**, **e**, **i** composition 3

Biological activity was investigated under artificial conditions in SBF (solution simulating cell-free body fluid). Since the silicon substrate does not react with the SBF solution, films on a silicon substrate (model substrate) were obtained using the centrifuging method. Films, regardless of their composition, are uniform (Fig. 6a–c) with a coating thickness for the SiO_2_–P_2_O_5_–CaO system is 86 nm, and for the SiO_2_–P_2_O_5_–CaO–MgO system, 62 nm. For the SiO_2_–P_2_O_5_–CaO–NaO system, the thickness is 78 nm.

The evaluation of the bioactivity of the obtained materials was studied in vitro by keeping the samples in cell-free simulation of SBF blood plasma for 14 days. The samples were placed in the model medium at a constant temperature of 37 ± 0.5 °C, pH 7.4. In this case, the solution was changed every day for 14 days. The composition of the SBF solution is described in ref (Kokubo et al. 1990).

After immersion in SBF solution, needle-shaped particles were observed on the surface of the samples and the dimensions reached above 30 nm (Fig. 6d–f). A change in surface morphology was seen when compared with the initial surface of the samples.

Regardless of the composition of the film coating on the surface of the samples, an increase in the concentration of Ca and P ions was observed after immersion in SBF (Fig. 6g–i). The presence of magnesium and sodium on the surface of the samples after immersion in SBF solution indicated the deposition of the components of SBF solution on the film surface.

An important role is played by both the absolute content of phosphorus and calcium in the coating, and their ratio. According to the X-ray microanalysis, in the first sample the Ca/P ratio is 0.9, and in the second sample, it is 1.41. Therefore, the introduction of magnesium has a beneficial effect on the bioactivity of the material.

**Fig. 7:**
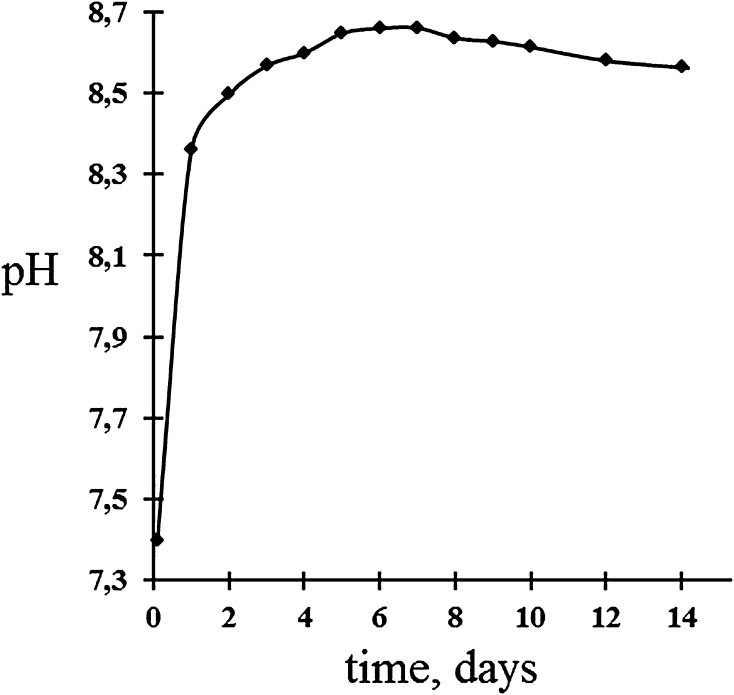
Change in solution pH within 14 days

As soon as the samples were immersed in a physiological solution, a rapid increase in the pH of the medium was observed on the 1st day, and later, the increase was not so significant (Fig. 7).

An increase in pH created a favorable atmosphere for the crystallization of the calcium–phosphate layer on the surface of the material (Xynos and Hukkanen 2000; Pereira et al. 1994).

The rapid release of alkali and alkaline earth ions from the solution, as well as an increase in pH, indicated a high reactivity of the samples under study. There were three stages in the process of complete bioactive bonding as follows (Pereira et al. 1994):

1. Rapid exchange of Na^+^ and Ca^2+^ with H^+^ or H_3_O^+^ from solution
  Si–O–Na^+^ + H^+^ + OH^−^ = Si–OH + Na^+^(aq) + OH^−^
  The pH of the solution increased as a result of H^+^ ions in the solution being replaced by cations
2. Soluble silica was lost in the form of Si(OH)_4_ in the solution, resulting from breaking of Si–O–Si bonds and the continued formation of Si–OH (silanols) at the solution interface:
  Si–O–Si + H_2_O = Si–OH + HO–Si
3. Condensation and repolymerization of an SiO_2_-rich layer on the surface, depleted in alkalis and alkali-earth cations.

The formation of a calcium**–**phosphate layer on bioactive materials and the release of soluble silicon and calcium ions into the surrounding tissues were key factors for the rapid connection of these materials with tissue.

Acceleration in formation of a calcium**–**phosphate layer on the surface of the material occurred with increasing pH (Pereira et al. 1994). An increase in pH indicated the dissolution of the cations from the surface of the material, which led to the formation of silanols, and promoted the formation of a calcium–phosphate layer. The leachability of cations from glass samples was a surface phenomenon, and the degree of ion exchange during the reaction in SBF depended on the pH value. In a slightly alkaline environment, the rate of these processes increased. Furthermore, the SBF pH was stabilized in all samples, which was associated with the absorption of calcium and phosphate ions from SBF to promote the formation of a calcium–phosphate layer on the surface of the samples.

## Conclusion

Thus, films of calcium–phosphate materials with the addition of sodium and magnesium ions were produced through sol–gel method. It is shown that the suitability of solutions for the preparation of films is limited to viscosity values that are in the range of 3.8–4.4 mm^2^/s. Solutions with magnesium ions at 20 wt% in the system increased the stability of the solutions up to 13 days. The introduction of sodium ion accelerated the processes in solution. The physicochemical processes of the formation of materials were studied, including the main stages: removal of physically and chemically bound water, combustion of alcohol and the products of thermo-oxidative destruction of ethoxy groups, and crystallization processes. The phase composition and structure of the films obtained were established at a temperature of 600 °C and above, and the crystalline forms of SiO_2_, CaSiO_3_, Ca_2_P_2_O_7_, and complex phosphates were fixed. In the system with the addition of magnesium ions, β-cristobalite SiO_2_ and stenfieldt Mg_3_Ca_3_(PO_4_)_4_ were detected; however, a crystalline sample was only obtained at 800 °C. In the system with sodium ions, chemical compounds Ca_5_(PO_4_)_3_Cl, NaCl, and SiO_2_ were determined. A uniform film coating was formed on the surface of the substrate. The introduction of sodium oxide into the SiO_2_–P_2_O_5_–CaO system increased the bioactivity of the materials obtained.
